# Manipulating Microbial Cell Morphology for the Sustainable Production of Biopolymers

**DOI:** 10.3390/polym16030410

**Published:** 2024-02-01

**Authors:** Vipin C. Kalia, Sanjay K. S. Patel, Kugalur K. Karthikeyan, Marimuthu Jeya, In-Won Kim, Jung-Kul Lee

**Affiliations:** 1 Department of Chemical Engineering, Konkuk University, 120 Neungdong-ro, Gwangjin-gu, Seoul 05029, Republic of Korea; vckaliaku@gmail.com (V.C.K.); sanjaykspatel@gmail.com (S.K.S.P.); karthikk1529@gmail.com (K.K.K.); inwon@konkuk.ac.kr (I.-W.K.); 2 Marine Biotechnology Division, National Institute of Ocean Technology, Chennai 600100, India; jeyambt@niot.res.in

**Keywords:** bacteria, biopolymers, biotechnology, cell division, cell morphology, polyhydroxyalkanoates

## Abstract

The total rate of plastic production is anticipated to surpass 1.1 billion tons per year by 2050. Plastic waste is non-biodegradable and accumulates in natural ecosystems. In 2020, the total amount of plastic waste was estimated to be 367 million metric tons, leading to unmanageable waste disposal and environmental pollution issues. Plastics are produced from petroleum and natural gases. Given the limited fossil fuel reserves and the need to circumvent pollution problems, the focus has shifted to biodegradable biopolymers, such as polyhydroxyalkanoates (PHAs), polylactic acid, and polycaprolactone. PHAs are gaining importance because diverse bacteria can produce them as intracellular inclusion bodies using biowastes as feed. A critical component in PHA production is the downstream processing procedures of recovery and purification. In this review, different bioengineering approaches targeted at modifying the cell morphology and synchronizing cell lysis with the biosynthetic cycle are presented for product separation and extraction. Complementing genetic engineering strategies with conventional downstream processes, these approaches are expected to produce PHA sustainably.

## 1. Introduction

Plastic products are used in almost all aspects of life and have become an integral part of our lives. Because of their unique thermochemical properties, such as their plasticity, adaptability, durability, and flexibility, they are convenient to use. Conventional plastics are derived from petroleum and natural gases. The high consumption rate of fossil fuels for their production has aggravated the energy crisis [1]. Being non-biodegradable, plastic waste accumulates in nature at a staggering rate, posing major management and environmental concerns [2]. Therefore, it poses a major threat to ecosystems and the environment. In addition, its negative impacts on human health cannot be ignored [3,4]. In 2020, the total amount of plastic waste was estimated to be 367 million metric tons. This number is anticipated to increase exponentially over the next few decades [5,6]. In principle, plastic waste can be managed through (a) recycling, which is not economically feasible, and (b) an energy recovery process, which emits greenhouse gases and toxic compounds, in addition to environmental pollution [7,8]. Therefore, strategies are needed to find alternatives to circumvent these issues.

The potential candidates envisaged to replace plastics are naturally biodegradable polymers. Based on biodegradability as the major criteria, the strong contenders are polycaprolactone (PCL), poly(butylene succinate), poly(ethylene succinate), poly(butylene adipate terephthalate, polyhydroxyalkanoates (PHAs), polylactic acid (PLA), starch, cellulosic esters, and proteins [9]. Of these, PHAs, PLAs, and starch are bio-based and -degradable polymers, which can be used for a wide range of biotechnological applications [10]. The various strategies for enhancing PHA production, include the genetic engineering of microbial cells to manipulate the cell morphology and size, modify metabolic pathways associated with PHA biosynthesis, downregulate unnecessary byproducts, and ease the recovery process [11,12,13,14,15,16]. In this article, the bioengineering of cell morphology and cell division machinery has been shown to have the potential to simplify downstream processing procedures and reduce production costs. In contrast to most of the published literature targeting sustainable PHA production strategies, the present article provides information on enhanced downstream process efficiency via exploiting genetic engineering to synchronize the processes of cell division, PHA biosynthesis, cell lysis, and product separation. The major emphasis is placed on developing methods for enhanced production and economic recovery for the sustainable production of biopolymers.

A bibliographic search was performed for research and review articles published in scientific journals and book chapters listed primarily on Scopus, PubMed, and Google Scholar, based on the following keywords: biopolymers, polyhydroxyalkanoates, inclusion bodies, cell morphology, cell division, genetic engineering, PHA biosynthesis, sustainability, down streaming, depolymerases, and recovery. The final text matter was written after critically reading and evaluating around 700 articles published primarily during the last ten years.

## 2. Biopolymers

Past research studies have focused on developing bioplastics based on renewable resources. Bioplastics and biopolymers can be produced from biomaterials of diverse origins [17,18]. The most used bioplastics are based on PHAs, PLA, and PCL. Other materials suitable for bioplastic production include starch, cellulosic esters, and proteins [9].

### 2.1. Polyhydroxyalkanoates

Polyhydroxyalkanoates are a large group of biodegradable thermoplastic polymers [19]. Many microbes that can produce PHA have been identified, including *Bacillus*, *Cupriavidus*, *Pseudomonas*, *Aeromonas*, engineered *Escherichia coli*, and *Halomonas* spp. [9,17,20,21,22,23]. Polyhydroxybutyrate (PHB) is a naturally produced biopolymer. PHB is also a homopolymer that is highly crystalline and brittle. These properties limit its range of biotechnological applications. In contrast, PHAs comprise more than 160 monomeric units [24]. PHAs are produced from pure chemicals and biowaste through fermentation under environmental stress conditions, primarily owing to the limitations of nitrogen, oxygen, phosphorus, and potassium. Pure microbial cultures and their consortia have been used to ferment diverse biowastes to produce copolymers of PHAs [22]. The diversity of monomeric units in copolymers of PHA confers them with properties like those of conventional plastics and has been demonstrated to have thermochemical properties suitable for applications in medicine, agriculture, and tackling diverse environmental issues [25,26,27,28]. A detailed description of these PHAs is presented in the following sections.

### 2.2. Polylactides

Polylactides are lactic acid (2-hydroxy propionic acid) polymers. Bacteria ferment carbohydrates to produce lactic acid. The biosynthesis of lactic acid is preferred because of the presence of L-stereoisomers in greater yields, leading to cheaper PLA production. These can be produced with diverse crystallinities, microstructures, and molar masses. However, they can also be synthesized chemically. Their synthesis can be completed through the polycondensation of lactic acid and polymerization of lactides via a ring-opening mechanism [29]. The major limitation is in the preparation and purification of pure lactic acid. During polymerization, certain undesirable side reactions take place. The presently available PLAs are based on linear macromolecules. For better physical features, there is a need to produce branched, dendrigrafic, or dendritic PLAs. It is difficult to produce high-molecular-weight PLAs with desirable mechanical properties, and the maximum molar masses have been limited to 6 × 10^4^ [30]. PLA is used for packaging and producing fibers and fabrics. As a blend, its use can be extended to implants, screws, nails, and plates for applications in the medical sector [28,31].

### 2.3. Polycaprolactones

Polycaprolactones are synthesized from crude oils. Although they are synthetic polymers, they are biodegradable, hydrophobic, and semicrystalline. Their crystallinity can be manipulated by regulating the low-molecular-weight alcohols. These excellent blends have low melting points and high rates of solubility, absorbability, and compatibility. Their viscoelastic and rheological properties are superior to those of other polymers. These characteristics enable their application in tissue engineering. However, their use in medical devices is limited because only a few fungal and bacterial species contain enzymes that degrade them [32]. These materials have excellent long-term degradation applications, such as in drug delivery through encapsulation. Efficient drug delivery systems can be complexed with polylactic acid-co-glycolic acid, PLA, cellulose acetate, butyrate, and propionate [33].

### 2.4. Other Polymers

Starch-based polymeric films are composed of 5–30% starch along with water or plasticizers (sorbitol and glycerol). Such starch-based biodegradable thermoplastics are both crystalline and amorphous. They can be produced easily in large quantities; however, they require high temperatures (91–180 °C) to melt and their biodegradation rate is limited to 30%. The starch-based bioplastic products include grocery bags, food and fruit packing trays, paper foam, egg boxes, and packaging electronic devices [34,35].

Cellulose is the most abundant naturally produced biodegradable polymer. Cotton linters and wood pulp are the major contributors to polysaccharide cellulose. Cellulose-based films are easy to produce, whereas bioplastics are difficult to make [36,37,38]. Cellulosic bioplastics are produced on an industrial scale as esters or ether derivatives [39]. The generally produced derivatives include nanofiber cellulose, cellulose nanocrystals, cellulose acetate, cellulose acetate butyrate, and biopolyethylene. There is a critical requirement for additives in the production of thermoplastics [36,38]. Cellophane, either transparent or pigmented, is commonly used to wrap candies and flowers, for lamination, and to pack food products such as coffee, cheese, and chocolate [28].

Protein-based polymers are heteropolymers of amino acids with unique characteristics, such as high mechanical strength and the ability to resist the diffusion of gases and aromatic compounds. Blending these polymers with keratin results in a mechanically strong, highly thermostable, and flame-resistant material. Biopolymers can be produced via physicochemical and thermoplastic processes [40,41]. Their availability in large quantities, high nutritional value, biodegradability, and use to prepare films make them highly desirable for the packaging industry [41]. Their use as matrices for the well-regulated release and immobilization of enzymes and their high-water retention capacity widen the scope of their application in agriculture, horticulture, and health. The time-resolved small-angle X-ray scattering technique allows in situ observations, particularly of molecular rearrangements and orientations [42].

Polyamide 11 is a biopolymer produced from renewable sources. This polymer has greater longevity than other biopolymers. Its unique thermochemical properties, such as its high stability and melting point (200 °C), make it suitable for manufacturing in industrial sectors. Its other desirable properties include its resistance to chemicals, water, oil, salts, fuels, and radiation. It has strong resistance to cracks and abrasion. Other features of this polymer include its low rigidity and poor resistance to volatile fatty acids and phenols, radiation, and heat. Its applications range from the aerospace and automobile sectors (electrical cables, water tubing, and natural gas piping) to other industries, such as footwear, metal badminton racket strings, coatings, and shuttlecocks. Although it is non-biodegradable, its recycling efficiency rate is high. Compared with polyamides, it is costly [41,43].

Spider silk is fabricated from renewable resources using spiders. It has unique mechanical properties such as a strength of up to 1.7 GPa (gigapascal) and an extensibility rate of up to 500%. It has a natural capacity to self-assemble into hydrogels, films, capsules, and spheres but has several limitations, including its poor solubility and storage and assembly abilities [44]. However, it is still suitable for manufacturing sports goods, ropes, textiles, robotic components, and composite materials. In the medical sector, its applications range from wound healing and the bridging of nerve defects to fascial replacements [39,45,46]. At present, the commercial-scale production of this biopolymer is limited by the following factors: (i) the cannibalistic nature of spiders, (ii) the farming of the spiders, (iii) the time-consuming nature of silk harvesting, and (iv) low yields. The silk protein spidroin is genetically expressed in microbes, such as bacteria, yeast, plants, and animals [36,46,47].

The demand for PHAs is increasing because of their diverse potential applications in agriculture, medicine, and the environment. The primary issues with PHAs are their production costs and the difficulties in recovering intracellularly produced biopolymers. These targets can be achieved through (i) bioengineering the morphology and size of microbes and (ii) downstream recovery. Different strategies are anticipated to contribute to the sustainable production of PHAs.

## 3. Sustainable Production of Biopolymers

The scope of the biotechnological applications of PHAs has been reported in the fields of medicine, horticulture, agriculture, and food, which has increased the demand for PHAs. However, their production on an industrial scale is restricted by their poor quality and the high cost of production using natural PHA-producing bacteria. In addition, there are issues with the polymers’ instability and the high variability in their thermomechanical properties [20,47,48]. Most research efforts have targeted the screening of naturally occurring microbes with high PHA yields, exploiting biowastes to produce copolymers [24,49,50]. Recently, researchers have shifted their focus to genetic engineering, genome mining, genome reduction, and genome editing [11]. For sustainable PHA production, there are a few possibilities that can be adopted. These proposals include modifying the cell morphology, downregulating the associated biosynthetic pathways, and focusing on the downstream process [12,13,14,15,16]. Here, we focused on genetic modifications to manipulate the cell morphology, size, and extraction of intracellularly produced PHAs.

Bioprocesses can be divided into two major groups—upstream and downstream [13]. Upstream metabolic processes are limited by the cost of the substrate, pretreatment, and energy input. Other important components are the microbial cell factories. The screening and selection of robust organisms that can resist environmental stress can be complemented by the genetic modification of the microbial strain [51]. Equally important are the costs and parameters of the downstream processes [52,53]. The recovery and purification of intracellular biomolecules through various physiochemical processes are more expensive than for extracellularly produced bioproducts [54]. Overall, for the sustainable production of polymers, it is important to develop methods for enhanced production and economic recovery [49].

### 3.1. Bioengineering of Microbial Cells

**Figure 1:**
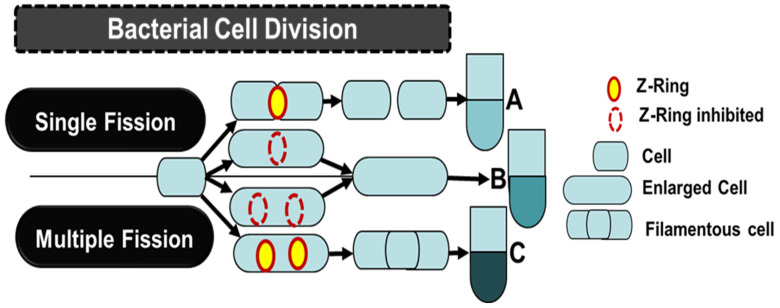
Diagrammatic representation of the effects of bioengineering cell division genes on the cell morphology and size: (A) binary cell division; (B) enlarged cell formation through inhibition of Z-ring formation via the overexpression of *sulA* or deletion or downregulation of genes involved in the biosynthesis of cell walls (*murA*, *murC*, *murD*, *murE*, *mraY*, *dxs*, *glmU*, *idi*, *pgi*); (C) filamentous cell formation via multiple fission through the deletion and downregulation of genes involved in the divisome (*ftsZ*, *ftsA*, *ftsW*, *minC*, *minD*, *pbp1*, *pbp3*, *sulA*, *slmA*, and *zipA*), hydrolases (*pbp5*, *pbp7*, *ampD*, *amiA*, and *amiB*), and rod complex (*mreB*, *rodZ*, *rodA,* and *pbp2*). Cell densities (A–C) vary by size and type [55,56,57,58,59,60,61,62,63,64,65,66,67,68,69].

The manipulation of microbes using genetic engineering and systems biology has been envisaged to be economical. Efforts have been focused on modifying the cell morphology and size (Figure 1) [55,56,57,58,59]. This strategy enables the greater accumulation of intracellular molecules, making the separation process convenient and economical [52].

#### 3.1.1. Cell Morphology 

The small size of bacterial cells makes their separation from the broth challenging. Furthermore, the rigid cell wall is a major limitation for the greater accumulation of inclusion bodies. These factors substantially increase the separation costs. A weak cell wall structure is likely to allow cells to expand, thereby providing more space for the storage of PHA granules. Large-sized cells can reduce recovery costs. Furthermore, to allow for the greater accumulation of intracellular biomolecules, enlarged cells can prove helpful. The primary focus of cell morphology is the modification of the cell shape and size [49]. This cell morphology engineering strategy showed that PHA production using enlarged bacteria is an effective technique for enhancing the product yield and easing downstream activities. 

Many genes are involved in cell wall synthesis and division, and those responsible for maintaining the cell shape are critical for the overall morphology (Table 1) (Figure 1) [60,61,62,63,64,65]. Several proteins are associated with these cellular characteristics. The cell wall component, peptidoglycan, is a network of cross-linked glycan chains [66]. It provides mechanical strength to the microbial cells, especially for withstanding environmental stresses. Many genes are involved in the biosynthesis of the cell wall (*murA* (encodes for UDP-*N*-acetylglucosamine enolpyruvoyl transferase), *murC* (encodes for UDP-*N*-acetylmuramate-alanine ligase), *murD* (encodes for UDP-*N*-acetylmuramoyl-L-alanine:D-glutamate ligase), *murE* (encodes for a ligase), *mraY* (encodes for phosphor-*N*-acetylmuramoyl-pentapeptide transferase), *dxs* (encodes for 1-deoxyxylulose-5-phosphate synthase), and *glmU* (encodes for N-acetylglucosamine-1-phosphate uridyltransferase)) [65], and the associated proteins include (i) the divisome (*ftsZ* (encodes for tubulin-like protein), *ftsA*, *ftsW* (encodes for a lipid II flippase)*, minC*, *minD*, *pbp1*, *pbp3*, *sulA*, *slmA*, and *zipA*, (actin-related proteins)), (ii) hydrolases (*pbp5*, *pbp7*, *ampD*, *amiA*, and *amiB*), and (iii) the rod complex (*mreB* (the cytoskeletal protein), *rodZ* (transmembrane protein), *rodA* (the transglycosylase), *pbp2* (penicillin-binding protein), and transpeptidase) [67,68,69]. The major advantages of these genetic manipulations are enhanced product accumulation and the ease of the downstream processes [55,70]. 

As the cell wall rigidity continues to limit PHB accumulation, manipulating the cell wall’s biosynthetic pathway remains a key goal. The cell rigidity can be manipulated by inserting the P*gltA* constitutive promoter for the *gltA* gene (which encodes for citrate synthase) before the genes responsible for cell wall synthesis. Using CRISPRi technology, the expressions of the following 10 genes involved in cell wall synthesis were downregulated: (i) *ftsW*, (ii) *dxs,* (iii) *glmU*, and (iv) *idi*, which encode for isopentenyl diphosphate isomerase; (v) *pgi* (encodes for phosphoglucose isomerase); (vi) *murA,* (vii) *murC*, (viii) *murD*, (ix) *murE*, and (x) *mraY*. In *E. coli* cells, the overexpression of the *sulA* gene resulted in Young’s modulus increases of 1.32- to 1.60-fold compared to the parent cells. Depending on the cell wall rigidity and thickness, the PHB accumulation rate was almost 4-fold (93%) in weakened cell walls compared to 25% in thickened cell walls [65].

#### 3.1.2. Cell Division 

Cell division via binary fission involves mass duplication and partitioning into two daughter cells via cytokinesis [71]. Inhibiting cytokinesis results in the formation of enlarged filamentous-shaped bacterial cells [72]. The most critical protein is FtsZ, which is responsible for forming a Z-shaped ring structure in the middle of the bifurcating cell. It interacts with other proteins to form a divisome [64]. Deleting the *ftsZ* gene inhibits FtsZ activity, leading to the abortion of the cell division process [73,74]. In addition, FtsZ protein interactions with other proteins encoded by *sulA*, *minC*, *minD*, *slmA,* and *EzrA* can help achieve higher PHA yields. The overexpression of *sulA* blocks FtsZ ring assembly and transforms the rod-shaped E. coli cells into filamentous cells shapes. This leads to the availability of a larger intracellular space [72,75]. This approach has been exploited to enhance the PHA yield in diverse bacteria (Table 2). In *E. coli*, filamentous cells showed 100% increases in PHB accumulation and total cell dry weight. An interesting feature of the cell enlargement strategy was the production of copolymers of PHA P(3HB-co-4HB) when using engineered *E. coli* harboring the *phaCAB* operon and the knockout of genes *sad* and *gabD*, as well as the essential genes *folK* and *ispH.* The resultant P(3HB-co-4HB) copolymer’s accumulation rate was greater than 78% of the cell dry weight. Thus, PHA facilitates recovery from the broth [75]. The overexpression of *minC* and *minD* is also instrumental in inhibiting FtsZ’s function [76]. Cell division enzymes (hydrolases) are necessary for daughter cell separation. The separation process is regulated by EnvC and NlpD, which regulate the amidase activity [77]. *E. coli* cells with deletions of *envC* and *nlpD* were found to inactivate amidase, resulting in their inability to separate daughter cells, leading to their filamentous shape [74]. This approach was further improved to obtain longer filamentous cells by overexpressing *sulA*, which inhibited the functioning of FtsZ. A switch from binary to multiple fission modes was observed in *E. coli* with a *minCD* deletion complemented by the overexpression of *sulA*. The mutants had 70% PHB storage capacity, which was higher than the 51% recorded with the wild type, along with a rate of 64% in filamentous cells growing in the binary fission mode. Thus, it supports the easy separation and greater recovery of PHB [74]. *Halomonas bluephagenesis* TD08 was engineered to overexpress *minC* and *minD* during the stationary phase of the cell cycle. The higher quantities of these proteins led to 1.4-fold longer cells, with the PHB content increasing from 69% to 82%, while the cells were 1.4-fold larger than those of the parent, attaining a cell size of a few hundred micrometers. Filamentous cells aggregate and settle in the fermenters within 12 h, facilitating cell separation without centrifugation or filtration [57].

*Pseudomonas* species are also well-known for their mcl-PHA-producing abilities. Initial efforts to manipulate the cell morphology did not enhance the mcl-PHA yield. Taking cues from other studies, many genes known to regulate the cell morphology, such as *nlpD* (peptidoglycan degradation) and *mreB* (cytoskeleton protein), as well as z-ring formation (*ftsZ*) and inhibition (*sulA*), were overexpressed in a *minCD* (regulates the z-ring location) knockout mutant of *Pseudomonas mendocina* NK-01, which increased the mcl-PHA yield by 45.62% (up from 0.28 to 0.41 g/L). This rate can reach up to 60.87% with the overexpressed *mreB* [78]. Apart from most studies focusing on the cell morphology and size, an additional feature that can complement these strategies and contribute to enhanced PHA yields is the manipulation of the PHA granule size. Phasin (PhaP), a protein located on the surfaces of PHA granules, regulates the granule number and size. The overexpression of *minCD* genes and deletion of *phaP* resulted in a lower number of PHA granules but enhanced the granular size. PHA granules of up to 10 μm were reported for the first time in *Halomonas bluephagenesis* TDH4-minCD-ΔphaP1. Thus, genetic engineering techniques have enabled the production of larger cells possessing larger PHA granules. In addition, the 4HB mol% in the PHA copolymer was 14% higher than that of the wild-type strain (Table 2) [16].

**Table 1:** Potential bacterial cell morphology and divisiome genes affecting polymer production.

Gene	Gene Product	Cell Function/Activity	Reference
* dxs *	1-deoxyxylulose-5-phosphate synthase	Cell wall synthesis	[65]
* glmU *	N-acetylglucosamine-1-phosphate uridyltransferase	Cell wall synthesis	
* murA *	UDP- * N * -acetylglucosamine enolpyruvoyl transferase	Cell wall synthesis	
* murC *	UDP-*N*-acetylmuramate-alanine ligase	Cell wall synthesis	
* murD *	UDP-*N*-acetylmuramoyl-L-alanine:D-glutamate ligase	Cell wall synthesis	
* murE *	Ligase	Cell wall synthesis	
*murJ*	Putative lipid II flippase	Regulates peptidoglycan incorporation to the septum	[67]
*ftsZ*	Bacterial fission ring formation protein	Recruiting divisiome proteins and z-ring stabilization	[64,73,74]
*ftsA*	Cell division protein	Divisiome	[69]
*ftsW*	Peptidoglycan glycosyltransferase, lipid II flippase	Divisiome	[65]
*ftsL*, *ftsN*, *ftsQ*	Cell division proteins	Divisiome	[67]
*sulA*	Cell division inhibitor protein	Divisiome, induces FtsZ inhibition	[72,75,78]
*slmA*	Nucleoid-associated FtsZ binding protein	Divisiome	[60]
*minC*	Z-ring positioning protein	Divisiome: actin-related proteins, inhibits FtsZ polymerization	[57,76]
*minD*	Z-ring positioning protein	Divisiome: actin-related proteins, recruits MinC	[57,76]
*envC*	Murein hydrolase activator	Divisiome	[74]
*zipA*	Integral inner membrane protein	Divisiome	[62]
*PBP1*, *PBP3*	Penicillin binding proteins	Divisiome	[68]
*envC*	Regulate amidase activity	Cell division	[68,77]
*nlpD*	Murein hydrolase activator, peptidoglycan degradation	Cell division	[74,77,78]
*mreB*	Dynamic cytoskeletal protein	Rod complex and cell division	[55,59,72]
*RodZ*	Transmembrane protein	Rod complex	[69]
*RodA*	Transglycosylase, lipid II flippase	Rod complex	[63]
*PBP2*	Penicillin binding protein, murein DD-transpeptidase	Rod complex, cell elongation	[68]
*gltA*	Citrate synthase	Manipulate cell rigidity	[65]
* idi *	Isopentenyl diphosphate isomerase	Cell wall synthesis	[65]
* mraY *	*Translocase 1,* phosphor-*N*-acetylmuramoyl-pentapeptide transferase	Cell wall synthesis	[65]
* pgi *	Phosphoglucose isomerase	Cell wall synthesis	[65]
*PBP5*, *PBP7*	DD-carboxypeptidases and DD-endopeptidases	Hydrolases	[61]
*ampD*, *amiA*, *amiB*	MurNAc-L-Ala amidases	Hydrolases	[61]

**Table 2:** Genetic manipulation of cell morphology for enhanced biopolymer production.

Organism	Gene Edited ^b^	Characteristics Affected	Impact on Polyhydroxyalkanoate ^c^ Production	Reference
*Escherichia coli* JM109SGIK	*sulA*	Transformation of rod to filamentous cell with larger internal space	PHB accumulation showed 100% increase	[75]
*E. coli* JM109SGIK	*sad*, *gabD*, *ispH folk*, and *sulA*	Transformation of rod to filamentous cell with larger internal space	Copolymers of PHA [P(3HB-co-4HB)] were 10% higher (78% in cell dry weight, CDW).	[75]
*E. coli* JM109SG (Δ*mreB*/pTK-*mreB*/pBHR68) ^a^	*ftsZ*, *mreB*, and *sulA*	Enlarged cell space due to reduced restriction on space. Larger volume to size ratio.	PHB ^d^ production was observed to increase from 5.72 g/L to (9.29 g/L, with a yield of 73.53% of CDW) ^e^ in a shake flask	[59]
*E. coli*	*envC* and *nlpD*	Switch from binary to multiple fission mode	PHB storing capacity enhanced from 51 to 70%	[74]
*E. coli* JM109	*ftsZ* and *mreB*	Enlarged cell volume	Enhanced PHB accumulations (up to 80%)	[73]
*E. coli* JM109	*ftsW*, *dxs*, *glmU*, *idi*, *pgi*, *murA*, *murC*, *murD*, *murE*, and *mraY*	Cell wall thickening	PHB accumulation of 93% in weakened cells and 25% in thickened cell walls	[65]
*Pseudomonas mendocina* NK-01	*ftsZ*, *mreB*, *sulA*, *minCD*, and *mreB*	Modified bacterial shape and growth pattern	Increased mcl-PHA ^f^ yield by 45.62% and up to 60.87%	[78]
*Halomonas bluephagenesis* TD08	*minCD*	Enlarged cells (1.4-fold longer than the parent)	PHB content enhanced from 69 to 82%	[57]
*Halomonas campaniensis* LS21	*ftsZ* and *mreB*	Enlarged cell morphology	Increase in PHB yield accompanied by normal growth	[72]
*H. bluephagenesis* TDH4-minCD-ΔphaP1	*phaP1*, *phaP2*, *phaP3*, and *minCD*	Bigger PHA granules and larger cell size	PHA granules up to 10 μm. PHA copolymer with 14% higher 4HB mol%	[16]

Note: a: *E. coli* strains were engineered using the *phbCAB* operon encoding PHA synthase, beta-ketothiolase, and acetoacetyl-CoA-reductase; b: genes (encoding enzyme): *dxs*, (1-deoxyxylulose-5-phosphate synthase); *envC*, (murein hydrolase activator); *ftsL*, *ftsN*, and *ftsQ*, (cell division proteins); *ftsW*, (peptidoglycan glycosyltransferase); *ftsZ*, (bacterial fission ring formation protein); *glmU*, **(**N-acetylglucosamine-1-phosphate uridyltransferase); *gltA*, (citrate synthase); *idi*, (isopentenyl diphosphate isomerase); *minC* and *minD* (Z-ring positioning protein); *mraY*, (*translocase 1*); murA, (UDP-*N*-acetylglucosamine enolpyruvoyl transferase); *murC*, (UDP-*N*-acetylmuramate-alanine ligase); *murD*, (UDP-*N*-acetylmuramoyl-L-alanine:D-glutamate ligase); *murE* (ligase); *mreB* (dynamic cytoskeletal protein); *nlpD* (murein hydrolase activator); *pgi*,(phosphoglucose isomerase); *sulA* (cell division inhibitor protein); c: polyhydroxyalkanoate; d: polyhydroxybutyrate; e: cell dry weight; f: medium chain length PHA.

#### 3.1.3. Cytoskeletal Protein

The bacterial cell morphology is also influenced by the cytoskeletal proteins encoded by the genes *mreB*, *mreC*, *mreD*, and *rodZ* [79]. The binding of MreB to the cytoplasmic membrane is regulated by PBP2, a cell wall biosynthesis enzyme [80]. The overexpression, deletion, and disruption of MreB and its associated proteins result in an abnormal cell morphology [81]. MreB deletion transforms rods into spherical cells. This transformation results in an enhanced volume-to-size ratio, enabling a larger space to accommodate intracellular molecules. Despite this benefit, the cell growth is drastically reduced, leading to a lower PHA yield. However, engineering the PHB biosynthetic pathway and expressing *mreB* in *E. coli* JM109SG at a low level helped restore the cell shape, improve the rigidity, and enhance the PHA yield by 60% (Table 2) [55,59]. These improved features can be exploited for the easy recovery of the cell biomass.

Growth retardation is a major limiting feature that is frequently encountered when engineering cell shapes. Thus, the expression of the genes *ftsZ* and *mreB* may play a vital role in optimizing these parameters [69]. *Halomonas campaniensis* strain LS21 with deletions of *ftsZ* or *mreB* genes was complemented with a plasmid expression system for these two genes. This enabled the mutant bacteria to grow even at 30 °C. Switching the growth temperature to 37 °C restricted the expression of the genes carried by the plasmid. This strategy resulted in greater PHB accumulation. The basic advantage of morphologically engineered cells is their rapid ability to settle to the bottom of the bioreactor and facilitate cell separation [72]. Synthetic biology techniques using clustered regularly interspaced short palindromic repeats (CRISPR) and their interference (CRISPRi) are efficient approaches for genome editing. The CRISPR system comprises the Cas9 protein and a single guide RNA (sgRNA) [82]. CRISPRi mutates the Cas9 protein, thereby allowing DNA binding to interfere with the transcription process [83]. CRISPR-based regulation was used to interfere with the expression of *ftsZ* and *mreB* in *E. coli*. This resulted in the production of long fat cells. The cell length and width were controlled using different sgRNAs. A wide range of morphologically diverse cells with enlarged cell volumes enabled the accumulation of PHB by up to 80% [73].

### 3.2. Complementary Extraction Processes

The extraction of intracellular molecules is an energy-intensive and expensive process. The major bottleneck is the breakdown of the cell walls, particularly in Gram-positive microbes [20,84]. Several studies have attempted to manipulate cell lysis and synchronize it with the substrate metabolism [20,85]. *E. coli* cells are susceptible to lysis because of their high concentrations of intracellular bioproducts. The PHA biosynthesis operon from *Cupriavidus necator* was expressed in *E. coli*, whereby the PHA yield increased to 70% of the total dry cell mass. The presence of *E. coli* biomass in a treatment with 0.2 N NaOH at 30 °C for one hour allowed the easy recovery of PHAs [86].

The bacteriophage (*E. coli* phage λ) holin operates by increasing the cell membrane’s permeability, whereas endolysin (lysozyme) metabolizes the cell wall [85]. Bacteriophage-based lysis takes place in the absence of Mg^2+^. PHA synthesis in engineered *E. coli* proceeds in the presence of Mg^2+^. The ion concentration is synchronized with the PHB production cycle, which at undetectable levels activates the phage lytic system and releases PHAs [85,87]. *E. coli* was engineered to trigger autolysis under stress by introducing a synthetic ribosome binding site and λ phage SRRz gene. The autolysis system (pSEVA331 plasmid) from *E. coli* was transferred into *H. campaniensis* LS21 cells to generate the *Halomonas* strain DL. This facilitated the economic recovery of PHA [88]. A similar approach was used to recover mcl-PHAs from *Pseudomonas putida* KT2440 [89,90]. The recovery rate of the PHAs was 94.2% [91]. As Gram-positive bacterial cells are more difficult to lyse, the *Bacillus amyloliquefacines* phage endolysin and holin system were expressed in *Bacillus megaterium* via the *E. coli*–*Bacillus subtilis* shuttle vector pX. Here, xylose was used for *yeoB* expression to synchronize cell lysis with the exhaustion of glucose and termination of PHA biosynthesis [20,92,93,94].

Bioengineered enlarged cells tend to settle due to gravitational forces, making the cell separation process easy and convenient to manage [55,75,95]. The efficiency of the cell-harvesting process can be improved using self-segregating and flocculating agents [96,97]. The bioengineering of *Halomonas campaniesis* LS21 through the deletion of the *etf* operon leads to a reduced cell surface charge and greater hydrophobicity. The net gain is attributed to self-flocculation, resulting in energy savings. In this way, the biopolymer’s productivity was enhanced by 1.8-fold to 0.33 g/L/h [98]. Solvent-based PHA extraction involves the usage of alcohols, ketones, dimethyl carbonates, esters, and cyclic carbonates [99]. The extraction of PHAs using halogenated solvents such as chloroform results in the excellent recovery of high-purity polymers from the biomass [99]. The major limitation is the large quantities required for extraction. Finally, a precipitation step is required to obtain high-purity PHAs. Here, “PHA anti-solvents” such as acetone, ethanol, heptane, hexane, or methanol are added in excess, which reduces the solubility of the PHAs in the solvent. This method is frequently used for PHA recovery from (i) bacterial co-cultures of *C. necator* DSM 428 (short chain length, scl-PHA producer) and *Pseudomonas citronellolis* NRRL B-2504 (medium chain length, mcl-PHA producer) [100] and (ii) a halophilic yeast *Pichia kudriavzevii* VITNN02 (scl-PHA, PHBHV) [101]. Other methods for PHA extraction use “green” solvents such as alcohols, ketones, cyclohexane, and esters, while a few of the novel solvents include acetone and non-cyclic ketones. “Green” solvents have been shown to recover (i) scl-PHA [102] and (ii) PHA-copolymers (poly(3HDD-co-3HD-co-3HO-co-3HHx) from *Pseudomonas chlororaphis* at room temperature [103]. Bioengineering cells to produce either scl- or mcl-PHA helps ease the solvent extraction process for scl-PHA from *C. necator* DSM 428 and the halophilic yeast *Pichia kudriavzevii* VIT-NN02 [101] and mcl-PHA from *P. citronellolis* NRRL B-2504 [100]. Because of its unique characteristics, such as its thermal stability, low inflammability, and low vapor pressure, PHA’s recovery rate is as high as 98% [104,105,106].

## 4. Perspectives

The replacement of non-biodegradable plastics with biopolymers has been envisaged as an ecofriendly and economical approach, especially for producing high-value products such as those required for the medical sector [107]. Biological processes have several major benefits over conventional chemical processes. The most critical requirement is mild environmental conditions, which saves on energy, potentially making them more economical. Although bioprocesses are highly specific and efficient, they are slower than chemical processes. The low quantity of bioactive molecules such as PHA produced per cell adds to the cost of the bioprocesses, making them uneconomical and unsustainable. Several mechanisms can be adopted to make biopolymer production sustainable. The production cost is primarily linked to the feed and the recovery of the biopolymer [20]. Several studies have focused on exploiting the bacterial ability to produce polymers, such as PHAs, from waste biomasses of diverse biological origins [17,18]. This also helps improve the thermochemical properties by producing copolymers of PHA [22,25,26,27,28]. However, the research efforts have focused on manipulating the bacterial morphology, particularly the cell size and morphology [11,55]. These modifications have proven beneficial for enhancing the efficiency of bioprocesses, resulting in accelerated growth, a higher cell density, and increased PHA accumulation [56,57,58,59]. These features simplify the downstream processes, helping to achieve higher yields and reduce costs [69]. In this article, we present an update on the genetic engineering of a few genes and their products responsible for the cell morphology [55]. Another potential approach that can facilitate PHA recovery is to use genetically modified organisms as supplements. Nuclease-producing genes from *Staphylococcus aureus* were engineered into *C. necator* and *Delftia acidovorans*. Nuclease enzyme reduced the viscosity of the broth containing disrupted cell biomass [108]. The *C. necator* PHA operon transformed the *E. coli* from a non-PHA producer to a producer state. The recombinant strain had poor cell integrity after accumulating PHA at up to 70% of the DCW. Simplified stirring of the 0.2 N NaOH broth at 30 °C for 1 h enabled the recovery of PHA with 95% efficiency [86]. These aspects are beneficial for separating the cells from the broth. Studies on genetic engineering to synchronize the cell lysis and substrate exhaustion process can help to avoid potential losses due to the onset of biodegradation within cells by depolymerases [84,85,109]. It must be emphasized that there other mechanisms, especially modifications of metabolic pathways such as biosynthesis, the availability of nutrients, energy generation, and the PHA granule size [12,13,14,15,16], can complement these approaches and help make the process economical and sustainable. 

Despite the potential benefits associated with cell morphology engineering leading to higher and more sustainable PHA production rates, there are quite a few challenges that need to be overcome. Efforts need to be made to improve the following processes: (i) shortening the cell cycle period; (ii) achieving higher growth rates and cell densities; (iii) modifying cell membranes to overcome the issue of low osmolarity rates; (iv) separating cells from broth by reducing the viscosity of the medium; (v) genetically regulating the termination of the PHA biosynthesis for product exhaustion, auto cell lysis, and the inhibition of PHA depolymerases; (vi) genetically modifying additional cell division genes and their associated proteins, especially hydrolases; (vii) searching for phages that can lyse bacterial cells; (viii) synchronizing PHA production with various processes for other value-added intracellular and extracellular products.

## 5. Conclusions

Plastics are some of the most popular and widely used synthetic polymers produced from natural gas and petroleum. Their non-biodegradable nature causes the accumulation of plastic waste, leading to environmental pollution. Given the limited fossil fuel reserves and the need to circumvent pollution problems, the focus has shifted to biodegradable biopolymers such as PHAs. Because they are produced by bacteria as intracellular inclusion bodies, their recovery and purification are critical for reducing the production costs. It is anticipated that bioengineering the cell morphology and cell division machinery can help simplify the downstream processes. Enlarged cell spaces allow for the greater accumulation of intracellular bodies and reduce the bioproduction cost. The bioengineering of the cell size to provide a larger intracellular space and the biosynthesis of PHA copolymers and other biomolecules can be complemented by engineering metabolic pathways and deleting the associated pathways that burden them. It must be realized that the TCA cycle is the main energy-generating mechanism in bacteria under normal environmental conditions. However, under stressed environmental conditions, especially with an abundance of C in the milieu, bacteria are provoked to store energy by curtailing the TCA cycle and diverting it towards PHA synthesis. The two pathways compete for acetyl-CoA and associated energy-generating reactions. Thus, it is recommended to regulate the energy metabolism, especially the flux of C, and prevent pathways that limit PHA synthesis. A few other potential mechanisms for enhancing PHA production are manipulating the PHA operon and restricting the expression of depolymerases. The overall benefit at the industrial scale is the sustainable production of biopolymers.

## Data Availability

Not applicable.
